# Development and Characterization of Yeast-Incorporated Antimicrobial Cellulose Biofilms for Edible Food Packaging Application

**DOI:** 10.3390/polym13142310

**Published:** 2021-07-14

**Authors:** Omar Mohammad Atta, Sehrish Manan, Abeer Ahmed Qaed Ahmed, Mohamed F. Awad, Mazhar Ul-Islam, Fazli Subhan, Muhammad Wajid Ullah, Guang Yang

**Affiliations:** 1 Department of Biomedical Engineering, Huazhong University of Science and Technology, Wuhan 430074, China; omarscience@yahoo.com (O.M.A.); sehrish_manan@hust.edu.cn (S.M.); abeer01ahmed01@yahoo.com (A.A.Q.A.); 2 Department of Botany and Microbiology, College of Science, Al-Azhar University, Assiut Branch, Assiut 71524, Egypt; 3 Department of Biology, College of Science, Taif University, P.O. Box 11099, Taif 21944, Saudi Arabia; m.fadl@tu.edu.sa; 4 Department of Chemical Engineering, College of Engineering, Dhofar University, Salalah 211, Oman; mulislam@du.edu.om; 5 Department of Biological Sciences, National University of Medical Sciences, Rawalpindi 46000, Pakistan; fazlisubhan93@gmail.com

**Keywords:** bacterial cellulose, yeasts, carboxymethyl cellulose, glycerol, antimicrobial activity, biocompatibility, edible film, food packaging

## Abstract

The unique properties and advantages of edible films over conventional food packaging have led the way to their extensive exploration in recent years. Moreover, the incorporation of bioactive components during their production has further enhanced the intrinsic features of packaging materials. This study was aimed to develop edible and bioactive food packaging films comprising yeast incorporated into bacterial cellulose (BC) in conjunction with carboxymethyl cellulose (CMC) and glycerol (Gly) to extend the shelf life of packaged food materials. First, yeast biomass and BC hydrogels were produced by *Meyerozyma guilliermondii* (MT502203.1) and *Gluconacetobacter xylinus* (ATCC53582), respectively, and then the films were developed ex situ by mixing 30 wt.% CMC, 30 wt.% Gly, 2 wt.% yeast dry biomass, and 2 wt.% BC slurry. FE-SEM observation showed the successful incorporation of Gly and yeast into the fibrous cellulose matrix. FTIR spectroscopy confirmed the development of composite films through chemical interaction between BC, CMC, Gly, and yeast. The developed BC/CMC/Gly/yeast composite films showed high water solubility (42.86%). The yeast-incorporated films showed antimicrobial activities against three microbial strains, including *Escherichia coli*, *Pseudomonas aeruginosa*, and *Saccharomyces aureus,* by producing clear inhibition zones of 16 mm, 10 mm, and 15 mm, respectively, after 24 h. Moreover, the films were non-toxic against NIH-3T3 fibroblast cells. Finally, the coating of oranges and tomatoes with BC/CMC/Gly/yeast composites enhanced the shelf life at different storage temperatures. The BC/CMC/Gly/yeast composite film-coated oranges and tomatoes demonstrated acceptable sensory features such as odor and color, not only at 6 °C but also at room temperature and further elevated temperatures at 30 °C and 40 °C for up to two weeks. The findings of this study indicate that the developed BC/CMC/Gly/yeast composite films could be used as edible packaging material with high nutritional value and distinctive properties related to the film component, which would provide protection to foods and extend their shelf life, and thus could find applications in the food industry.

## 1. Introduction

In addition to the parental role of food as a commodity for nutrition, presently, food is also considered as a regional or cultural ambassador. The food sector should maintain the sensory properties of foodstuffs such as taste, texture, and smell for maintaining its quality and provide protection against spoilage. Better food management would lead to the consumption of fresh and healthy food [1]. In order to satisfy the environmental standards, the food industry must extend the food packaging to sustainable and biodegradable goods [2]. Food packaging is defined as the coating or enclosing of food items within some bioactive materials with the aim to prevent them from physical, chemical, and biological contaminations, and thus increasing their shelf life [3]. Food packaging plays a vital role in food safety and preservation. For instance, the usage of edible films and food coatings have been shown to improve food safety, add value to edible polymer products, and minimize the use of non-degradable packaging materials such as plastics. The edible film is distinguished from food products during the food production and implementation process. For example, the membranes are dried and used to package a product or make bags, while edible coatings are usually added outside in liquid or dry form [4]. The edible films protect the consumable food products from deterioration by slowing down the drought and providing selective barriers to moisture, breathing suppression, gases such as oxygen and carbon dioxide, improving texture, helping to keep the volatile compounds, and preventing microbial growth on food surface [5].

Cellulose, as the most abundant biopolymer, is obtained from plants [6,7,8], microorganisms [9,10], animals, and some algae [11], as well as synthesized enzymatically [12,13,14]. Among the different sources, the cellulose obtained from bacteria, known as bacterial cellulose (BC), is receiving immense consideration due to its nanofibrillar three-dimensional (3D) network structure, purity, and unique structural, physico-chemical, mechanical, thermal, and biological features. Due to such features, BC is receiving immense consideration in the biomedical [15,16,17], additive manufacturing [18,19], environment [20,21], and food sectors [22,23]. BC is practically untapped for constructive or intelligent use as a food packaging material. Some work has been confirmed to use BC as an effective food packaging material where its composites with different nanomaterials demonstrate in vitro antimicrobial activity against certain microorganisms [24,25]. Earlier studies reported that BC could be cost-effectively produced and used in the manufacturing of edible membranes [26,27]. The addition of additives such as glycerol (Gly) further enhances the softening performance of BC films, thus contributing to increasing the elongation in fracture strength and response to the applied external pressure [28]. Unlike BC, which is insoluble in water and common solvents, carboxymethyl cellulose (CMC), as the water-soluble derivative of cellulose, is widely used in many food, pharmaceutical, laundry, and other industries [29]. It has desirable properties such as water solubility, oxygen transfer, fat resistance, high viscosity, and being odorless, tasteless, non-toxic, insensitive, clean, clear, and flexible [30], thus making it a suitable candidate for food packaging.

For edible and healthy foods, preserving the quality during the packaging period is a major challenge. Moreover, the newly emerging outbreaks necessitate the development of alternative packaging approaches which could preserve the quality and freshness of food while the food should remain healthy during the packaging period. All such challenges require the development of new types of active and non-toxic antimicrobials for food packaging applications [5,31]. To this end, the use of active materials such as yeast could be an effective approach in developing bioactive food packaging materials. The potential of yeast in preventing food decay has already been established over the last couple of decades. The incorporation of yeast into the packaging material could impart antimicrobial activity as well as enhancing its nutritional value and serving as the probiotic. For instance, the yeast *Meyerozyma guilliermondii* is non-pathogenic and demonstrates good antimicrobial activity, and is a rich source of vitamins and proteins [32,33]. The direct incorporation of *M. guilliermondii* or its metabolites to the packaging material imparts it with antimicrobial activity, and thus contributes to increasing the shelf life of food [34,35]. On the other hand, the common brewery yeast (i.e., *Saccharomyces cerevisiae*), although is a good source of important minerals, vitamins, and proteins of B complexes, unlike *M. guilliermondii*, lacks antimicrobial activity, and thus is not a preferred choice for food packaging applications. Different phenotypes of yeast have high efficacy in the management of pathogenic fungi, causing post-harvest infections, and providing protection to orange and lemon [36,37]. Yeast is also reported to prevent the growth of foodborne pathogens [38]. Studies have shown that the addition of probiotic bacteria to food packaging films and coatings enhances their survival rate and that the combination of bacterial strains and antimicrobial properties improves the shelf life of the packaged food items [39,40].

The present study is aimed to develop active food packaging by utilizing BC, CMC, and Gly with yeast as the antimicrobial agent. We investigated the impact of the addition of yeast on physico-chemical, mechanical, and thermal features as well as biological (i.e., antimicrobial and biocompatibility) properties of BC/CMC/Gly composite films.

## 2. Materials and Methods

### 2.1. Materials

Chemical reagents, including glucose, yeast extract, tryptone, sodium phosphate, citric acid, hydrochloric acid, and microbiological media, were purchased from Sigma Aldrich (St. Louis, MO, USA). Other chemical reagents, such as sucrose, Na_2_HPO_4_, (NH_4_)SO_4_, and MgSO4, were purchased from Sinopharm Chemical Reagents Co., Ltd. (Shanghai, China). All reagents used were of analytical grade and used without further processing and purification unless otherwise stated. All aqueous solutions were prepared using deionized distilled water.

### 2.2. Microbial Strains

The BC-producing *Gluconacetobacter xylinus* (ATCC53582) strain was obtained from the General Group of Microbiological Culture in China (Beijing, China). The yeast *Meyerozyma guilliermondii* (MT502203.1) was obtained from the Mycological Centre of Assiut University (Assiut, Egypt).

### 2.3. Bacterial Cellulose Production and Purification

BC hydrogels were statically produced by the *G. xylinus*, according to our previously reported study [41]. Briefly, few colonies from the agar plate culture of *G. xylinus* were inoculated into the liquid HS medium (pH 5) containing 20 g/L glucose, 5 g/L yeast extract, 5 g/L peptone, 3.4 g/L disodium phosphate, and 1.5 g/L citric acid. The microbial cell culture was incubated statically at 30 °C for 7 to 10 days. The BC hydrogels were harvested from the air–liquid interface and treated with 0.3 N NaOH, and autoclaved for 15 min at 121 °C and 15 psi to kill any live bacterial cells. The dead cells and residual medium components were removed through the repeated washing of BC hydrogels until the pH of the medium became neutral and finally stored in distilled water at 4 °C for further use.

### 2.4. Preparation of BC Slurry

The BC hydrogels were cut into small pieces and then mechanically blended for 1 h using a kitchen blender (MD-326S). The slurry obtained was collected and stored at 4 °C for further use.

### 2.5. Yeast Biomass Production

Yeast biomass was produced by culturing the *M. guilliermondii* on agar plates (pH 5) containing 20 g/L malt extract, 5 g/L peptone, and 20 g/L agar, according to a previously reported study [42]. Yeast biomass was harvested at the log phase and transferred to deionized sterilized water. The biomass was separated via centrifugation at 7000 rpm for 10 min. The obtained yeast biomass was air dried at 50 °C and stored at 4 °C for further use.

### 2.6. Preparation of BC-Based Composites Films with CMC, Gly, and Yeast

The composite films comprised 30 wt.% CMC, 30 wt.% Gly, 2 wt.% yeast, and 2 wt.% BC were prepared ex situ. First, 30 g of CMC was dissolved in 100 mL of distilled water followed by the addition of the desired amount of BC slurry equivalent to 2 wt.% dried cellulose, and finally 30 wt.% Gly. All reagents were mixed on a magnetic stirrer. Finally, 2 wt.% dried yeast was added, and the mixture was placed in a vacuum jar for the removal of bubbles and then placed on a rack and casted. The plates were held at 45 °C overnight, and finally, the prepared films were removed from the plates (Borosilicate Glass Petri Dishes, 20 cm × 2 cm, Wuhan, China). Films of the same concentrations were prepared with and without yeast. The thickness of the films was measured with a digital thickness gauge (CH-1-ST. Shanghai, China) by taking measurements from at least five different positions, and the values were averaged.

### 2.7. Characterization

#### 2.7.1. Solubility and Moisture Content

The solubility and moisture content of the films were determined as reported previously [43]. Briefly, 20 mm × 20 mm pieces of BC/CMC/Gly/yeast film were first completely oven dried at 110 °C and then immersed in 20 mL of distilled water for 3 h at 60 °C. Thereafter, the mixture was poured on a nylon cloth and filtered. The wet BC/CMC/Gly/yeast films were then washed with 10 mL of distilled water and oven dried again at 110 °C for 24 h. The dried films were then weighed. All treatments were performed in triplicate. The water solubility (WS) was determined as the ratio of the original dry weight (W_o_) and dry weight after immersion in water and drying (W_f_) and was calculated by using Equation (1).

(1) $$\mathrm{WS}\left(\%\right)=\frac{\mathrm{Wo}-\mathrm{Wf}}{\mathrm{Wo}}\times 100$$

Similarly, the water content (WC) of the films was determined as the film weight before drying (W_b_) and after drying (W_o_) and calculated by using Equation (2).

(2) $$\mathrm{WC}\left(\%\right)=\frac{\mathrm{Wb}-\mathrm{Wo}}{\mathrm{Wb}}\times 100$$

#### 2.7.2. Mechanical Testing

The mechanical properties of the BC/CMC/Gly/yeast film, including the tensile strength and elongation at break, were determined by using a 50 kg (Transcell Scale Co. Ltd., Chicago, IL, USA) compressive testing device SANS CMT4000 (MTS Industrial System Co., Ltd., Shanghai, China), as reported previously [44,45]. The films were cut into 1.5 cm × 10 cm strips. In addition to an initial separation of 5 cm, the films were suspended and dismantled by 25 mm/min. The tensile strength was determined by dividing the average resting force (reading from the tool or chart) by a transverse film region (N/m^2^ = Pascal). The percentage extension at rest was based on a longer duration relative to the original length of the film. The elongation at break was calculated by using Equation (3) [46].

(3) $$\mathrm{Elongation}\;\mathrm{at}\;\mathrm{break}\;\left(\%\right)=\frac{L\;-{\;L}_{0}}{L_{0}}\times 100$$

where L and L_0_ are the final and initial gauge lengths, respectively.

#### 2.7.3. Field-Emission Scanning Electron Microscopy

The surface morphology of freeze-dried pristine BC, BC/CMC, BC/CMC/Gly, and BC/CMC/Gly/yeast films was studied through a field emission scanning electron microscope (FESEM, NovaNanoSEM450, and FEI, Hillsboro, OR, USA). Briefly, the films were mounted on double sided tape on aluminum stubs and coated with a gold layer (40–50 nm) before SEM observation.

#### 2.7.4. Fourier-Transform Infrared Spectroscopy

The chemical interaction between BC, CMC, Gly, and yeast in the composite film was determined through Fourier transform infrared spectroscopy (FTIR) (VERTEX 70, Bruker, Germany). The spectra for all substances were recorded in the spectral range of 500 to 4000 cm^−1^ with a resolution of 0.5 cm^−1^ [47].

#### 2.7.5. Thermogravimetric Analysis

The thermogravimetric analysis (TGA) of pristine BC, BC/CMC/Gly, and BC/CMC/Gly/yeast films was performed to determine their thermal stabilities as a function of changes in mass with the changing temperature. The TGA of individual components and composite films was carried out using a thermogravimetric/differential analyzer (Q50 Thermo balance, USA). Thermograms were obtained in the temperature range of 30–600 °C under a nitrogen atmosphere with a temperature increase of 20 °C/min.

### 2.8. Antibacterial Activity

The antibacterial activity of the BC/CMC/Gly/yeast composite film was determined against *E. coli*, *S. aureus*, and *P. aeruginosa* via the disc diffusion method as reported previously [47,48]. First, all microbial strains were cultured on nutrient agar medium or yeast peptone dextrose (YPDA) medium, and subsequently, 1 cm diameter sterilized and dried disc samples were placed on the culture plates and incubated for 24 h at 37 °C. After incubation, the inhibition zones were measured for all samples.

### 2.9. Biocompatibility Evaluation

The biocompatibility of the BC/CMC/Gly/yeast composite film was determined for NIH-3T3 cells (mouse embryo cell line). Briefly, the cells were cultured in high glucose culture flasks (4.5 g^−1^) containing L-glutamine and pyruvate (110 g^−1^), DMEM, 10% FBS supplement (GIPCO, Waltham, MA, USA), and 1% penicillin/streptomycin, and incubated in 5% CO_2_ at 37 °C. The medium was changed every 2–3 days, and the cells were transferred every 3–4 days. For biocompatibility assessment, the BC/CMC/Gly/yeast films were placed in a 96-well microplate, seeded with 1 × 10^4^ cells/well, and incubated in a 5% CO_2_ incubator for 24 h. After incubation, the samples were washed three times with PBS and transferred into a fresh DMEM growth medium containing MTT (3-(4,5-dimethyl-2-thiazolyl)-2,5-diphenyl-2H tetrazolium bromide, 5 mg/mL) reagent at 10:1. The samples were incubated again at 37 °C for 4 h. Thereafter, the medium was removed, followed by the addition of formazan and 150 mL of DMSO (dimethyl sulfoxide). Finally, the absorption was measured at 570 nm by using a multi-scan spectrophotometer (Tecan, infinite F50).

### 2.10. Fruit Packaging via Dipping Method

The fruit packaging ability of BC/CMC/Gly/yeast composite films was determined by the dipping method, as reported previously [49]. Fruits, including tomatoes and oranges, were obtained from a local supplier (Wuhan, China). First, the fruits were sterilized for 2 min at commercial maturity with 200 ppm NaClO solution and allowed to air dry. Thereafter, the fruits were dipped in the BC/CMC/Gly/yeast solution for 2 min. All samples were divided into four groups according to different temperatures: the 6 °C (refrigerator), 30 °C and 40 °C (incubator), and 20–25 °C (room temperature) group. Each treatment group comprised three different samples, including the uncoated, BC-coated, and BC/CMC/Gly/yeast-coated fruit samples, and each sample was used in triplicate. During the experiment, the uncoated fruits were used as the control for every treatment. During the incubation period, the freshness of fruits was assessed for sensory features such as odor, color, dryness, and contamination, as reported previously [50,51]. Briefly, the sensory features of fruits were assessed by a panel of ten judges according to a scale of 1–10 with 1–2 = very poor, 3–4 = poor, 5–6 = fair, 7–8 = good, and 9–10 = excellent. A score of 5 was used as the cutoff value for product acceptability, according to a previous report [52].

### 2.11. Statistical Analysis

Each experiment was performed in triplicate, and all data are presented as mean ± standard error of the mean. A comparison between the control and treatment groups was carried out by the Student’s *t*-test using SPSS 22.0 software (IBM, Armonk, NY, USA), and the difference was considered statistically significant at * p < 0.05 or ** p < 0.01.

## 3. Results and Discussion

### 3.1. Preparation, Appearance, Moisture Content, and Water Solubility of BC/CMC/Gly/Yeast Composite Films

Carboxymethyl cellulose is easily soluble in water due to its polyanionic nature and is thus commonly used in the preparation of hydrocolloidal cellulose formulations [53]. In contrast, pristine BC is insoluble in water and common organic solvents [54,55]. Therefore, a slurry prepared through the mechanical grinding of BC was used instead of a membrane or a hydrogel in the preparation of the composite films. Furthermore, the use of antimicrobial material requires the selection of a concentration equal to the minimum inhibitory concentration (MIC) to avoid any cytotoxic effect. Therefore, the MIC of different concentrations of dry yeast biomass was determined (data not shown), which was equal to 2 wt.% yeast dry biomass. The ex situ preparation method resulted in the formation of 0.18 ± 0.039 cm and 0.17 ± 0.021 cm thick BC/CMC/Gly and BC/CMC/Gly/yeast films, respectively. A naked-eye observation showed that the BC/CMC/Gly/yeast composite films did not contain any fragile areas or bubbles and pores.

The moisture content results show that the addition of 30 wt.% Gly into the BC/CMC matrix increased the moisture content of the film from 9.72% to 30.11% (Table 1). This result is in agreement with a previous report where the addition of Gly into the quince seed mucilage-based edible film increased the moisture content as well as the vapor permeability and oxygen permeability of the films [56]. In another study, the addition of up to 10 wt.% Gly into the potato starch-based films greatly contributed to enhancing the permeability of the films to water and oxygen; however, a further increase in Gly content negatively affected the stability of the films, which could be due to the enhanced moisture content of the film. It has been reported that an increased moisture content decreases the barrier capability of the films and vice versa [57]. In the present study, the incorporation of yeast into the BC/CMC/Gly film (i.e., BC/CMC/Gly/yeast) decreased the moisture content of the film to 23.66%. This decrease in moisture content could be attributed to the discontinuities in the film matrix due to the presence of yeast cells, which make the film more open to mass transfer. However, considering the small size of the yeast cells, the decrease in moisture content is moderate. These observations are in accordance with a previous study, where the introduction of microorganisms into the matrix of sodium caseinate/methylcellulose composite film decreased the moisture content [58]. However, it should be noted that the introduction of different microorganisms into the film matrix may not necessarily decrease the moisture content. For instance, the introduction of microorganisms with hydrophilic surface properties may retain more water and lead to an increase in the moisture content of the film. Moreover, the addition of Gly and yeast into the BC/CMC matrix enhanced the water solubility of the films. The solubility of the polymer is an important factor for edible food packaging applications. It is anticipated that the hydrophobic compounds lower the solubility while the hydrophilic compounds enhance the solubility of the films [59]. Cellulose is hydrophilic in nature and is insoluble in common solvents because of long rigid chains and the presence of abundant free hydroxyl (OH) groups which form strong intra- and intermolecular hydrogen bonding between the chains [60]. The solubility of BC/CMC films in water was only 22.28%. The cellulose structure can be used by water, and the intermolecular interactions between BC and CMC can be broken. Therefore, the low soluble BC-based edible films were able to dissolve CMC in water only, and the remaining solid comprised split BC fiber. The solubility was increased to 39.54% and 42.86% with BC/CMC/Gly and BC/CMC/Gly/yeast, respectively, which could be attributed to the enhanced hydrophilic character of cellulose [59].

### 3.2. Mechanical Properties of BC/CMC/Gly/Yeast Composite Films

**Figure 1:**
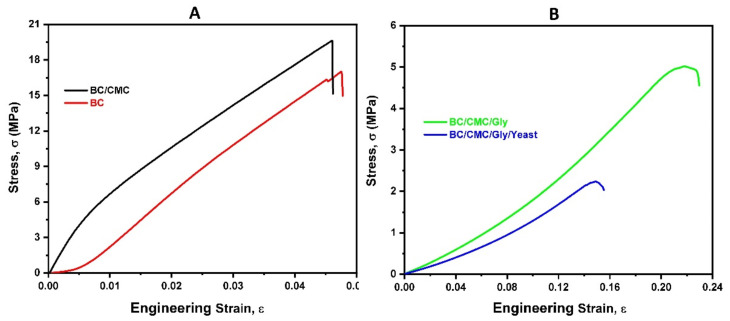
Stress–strain curves of (**A**) pristine BC and BC/CMC composite film and (**B**) BC/CMC/Gly and BC/CMC/Gly/yeast composite films.

The mechanical features, including the tensile strength and elongation at break of the composite films, were determined relative to pristine BC (reference), and the results are shown in Table 2 and Figure 1. It was expected that the addition of Gly and yeast might influence the flexibility of BC. The results showed that compared to the control (BC), the addition of yeast and Gly into the BC/CMC films resulted in decreasing the tensile strength and increasing the elongation at break. Interestingly, the addition of Gly to BC/CMC increased the elongation at break. These results are in agreement with a previous study, where the addition of 25–50 wt.% Gly decreased the tensile strength and increased the elongation at break of quince seed mucilage-based films [56]. In another study, the addition of up to 10 wt.% Gly into the potato starch-based films decreased the tensile strength; however, it did not significantly affect the elongation at break. In the present study, the addition of yeast into the matrix of BC/CMC/Gly film slightly decreased the tensile strength and elongation at break. The increased elongation at break of the BC/CMC/Gly/yeast composite film relative to pure BC and BC/CMC films could be attributed to the plasticizing behavior of Gly. This behavior is in agreement with a previous study where the addition of Gly and CMC greatly contributed to enhancing the plasticizing behavior and flexibility of the composite material [28].

### 3.3. Morphology of BC/CMC/Gly/Yeast Composite Films

**Figure 2:**
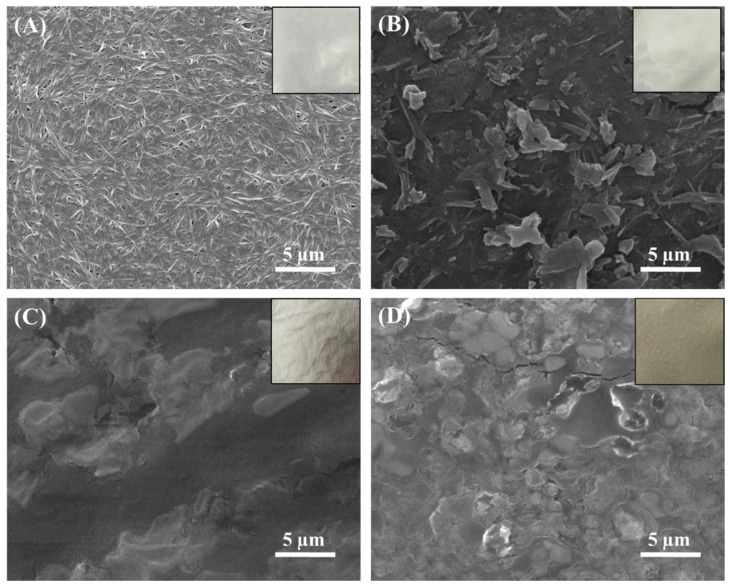
FE-SEM micrographs of surfaces of (**A**) pristine BC, (**B**) BC/CMC, (**C**) BC/CMC/Gly, and (**D**) BC/CMC/Gly/yeast films. The inset images show the respective freeze-dried samples.

The SEM micrographs of pristine BC and BC-based composite films are shown in Figure 2, while the respective digital photographs of all samples are provided as insets in Figure 2. As expected, the pristine BC exhibited a typical 3D reticulated fibrous network structure with randomly distributed fibers (Figure 2A). In contrast, the SEM micrograph of the BC/CMC composite film showed a high packing density of cellulose fibrils and the presence of many cracks on its rough and porous surface (Figure 2B). These observations are in accordance with a previous report [61]. This morphology of the BC/CMC composite film could be attributed to the large molecular size of the non-homogeneously dissolved cellulose fibrils. The SEM micrographs of the surface morphology of the BC/CMC/Gly film (Figure 2C) showed no obvious cracks, breaks, or openings, indicating no detrimental effect of Gly as a plasticizer, which is in accordance with a previous report [56]. In fact, the BC/CMC/Gly (Figure 2C) and BC/CMC/Gly/yeast (Figure 2D) composite films exhibited strong, smooth, and more homogeneous fibrous surface. This indicates that the incorporation of glycerol and yeast reduced the surface roughness, which in turn would affect the physico-mechanical and biological features of the composite films. The SEM results are in accordance with some previous studies, which show that the microstructural surface of BC-based composites with Gly [62] and polyvinyl alcohol [63], chitosan [64], and other materials show compact and rough surface morphologies.

### 3.4. Chemical Properties of BC/CMC/Gly/Yeast Composite Films

**Figure 3:**
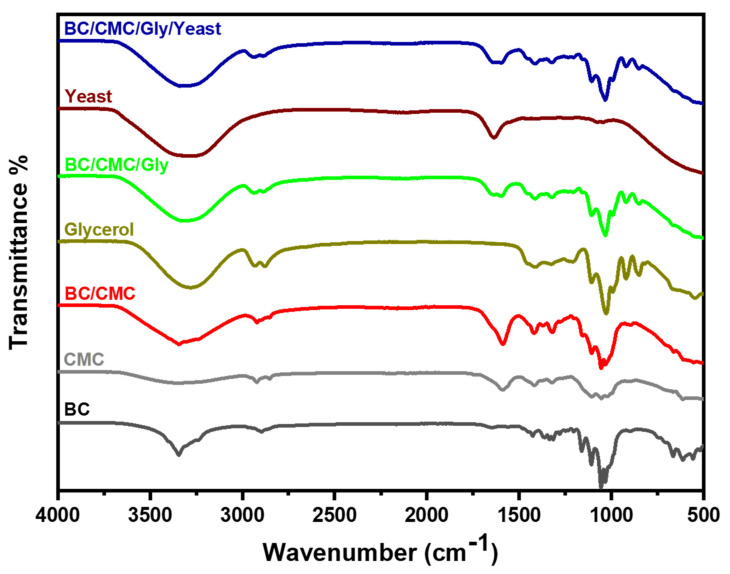
FT-IR spectra of pristine BC, CMC, BC/CMC, glycerol, BC/CMC/Gly, yeast, and BC/CMC/Gly/yeast.

FTIR is an important spectroscopic technique that gives information about the presence of different functional groups and the nature of chemical interaction between such groups in a molecule or a compound. In the present study, FTIR spectroscopy was carried out to investigate the chemical synthesis of BC/CMC and BC/CMC/Gly, as well as the integration of yeast into the latter composite. FTIR analysis was carried out both for individual components, including pristine BC, CMC, Gly, and yeast, as well as the composites, including BC/CMC, BC/CMC/Gly, and BC/CMC/Gly/yeast, and their characteristic spectra are shown in Figure 3.

The FTIR spectrum of pristine BC showed the characteristic peaks of cellulose [65,66], thus confirming the purity of BC synthesized by *G. xylinum* and the effectiveness of post-synthesis purification techniques such as treatment with 0.3 N NaOH and repeated washing with distilled water. Specifically, the FTIR spectrum of pristine BC showed characteristic peaks at 3440 cm^−1^, 2926 cm^−1^, 1650^−1^, 1300 cm^−1^, and 1440 cm^−1^ for O-H stretching, C-H stretching vibration, symmetrical CH stretching, C-H deformation, and CH_2_ deformation, respectively. This characteristic peak pattern of pristine BC was used as a reference. In the FTIR spectrum of BC/CMC, the broadening of the peak between 3500 cm^−1^ and 3100 cm^−1^ indicates the strengthening of hydrogen bonding compared to pristine BC; however, this band was relatively less broad than CMC alone, indicating a slight decrease in the strength of hydrogen bonding. These results are in agreement with a previous study [67]. Furthermore, the BC/CMC composite showed an extra band at 1631 cm^−1^, which is attributed to the stretching vibration of the carboxylate group formed in the composite, which is in accordance with a previous report [67]. This peak was present in the spectra of all samples, except pristine BC and Gly, although with different intensities. In conjugation with the bonds and aliphatic bending vibrations, the peak for vibration activity appeared at 3000 cm^−1^ for fatty acid vibration activity in the FTIR spectra of pure Gly and the BC/CMC/Gly composite film [68,69]. Similarly, the peak for the C-H group was present in all films between 2894–2925 cm^−1^ except pure yeast. A characteristic peak appeared at 1700 cm^−1^ in the spectra of pure yeast and the BC/CMC/Gly/yeast composite film for the vibration absorption band of the ester carbonyl C=O group, indicating the chemical interaction of yeast with the components of the composite film. However, unlike the characteristic peaks of BC and CMC, the peaks for yeast and Gly did not appear clearly in the spectrum of BC/CMC/Gly/yeast composite films and were largely overlapped by the signals of the BC/CMC composite film. These observations are in accordance with a previous report [70]. The FTIR results show the existence of the essential chemical interaction of cellulose backbone with other components in the composite films, although the peak intensity and their position varied slightly, indicating the formation of chemical bonding between the components of the composite films and strengthening or weakening of original chemical bonding of the individual components.

### 3.5. Thermal Stability of BC/CMC/Gly/Yeast Composite Films

**Figure 4:**
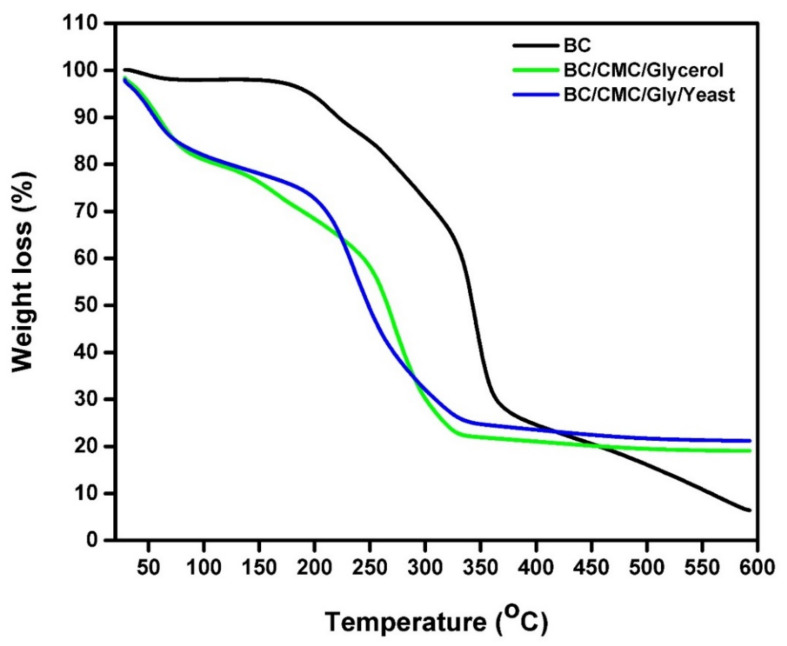
TGA curves of BC, BC/CMC/Gly, and BC/CMC/Gly/yeast films.

All substances possess their own thermal degradation temperature. The TGA of the composite analysis was carried out to determine their thermal stability at elevated temperatures. The thermal decomposition profiles of the samples are shown in Figure 4, which display two degradation regions: a region showing the weight loss due to dehydration and the other region showing the weight loss due to the decomposition of cellulose glycosyl units, according to previous research [65,71].

The thermogram of pristine BC shows a 2% decrease in weight up to an increase in temperature to 100 °C, which is attributed to the evaporation of water. Due to the use of dried BC films for TGA analysis, the hydrophilic nature helps absorb moisture. Moreover, the interlayer water molecules also contributed to the observed weight loss [72]. In contrast, the composite films showed a higher weight loss. For instance, the BC/CMC/Gly and BC/CMC/Gly/yeast composite films showed a 20% and 17% weight loss, respectively, during the first degradation step at 90 °C. This increased weight loss could be attributed to the fact that the films containing yeast or Gly did not dehydrate entirely in comparison with the film without Gly or yeast. In the second stage of degradation, around 70% weight loss was observed for pristine BC. Its degradation began at about 210 °C and continued until the cellulose chains were completely decomposed at about 350 °C. On the other hand, the second degradation step for BC/CMC/Gly/yeast started between 240 °C to 260 °C and continued until 330 °C when cellulose chains were broken, which resulted in a 75% weight loss. Studies have shown that the main BC cellulose skeleton degrades up to 300 °C [65,73]. These results show that the addition of Gly and yeast slightly reduced the thermal degradation behaviors of the composite film, thus indicating the improved thermal stability of BC at elevated temperature, which is useful for the sterilization of packaging material. The thermogravimetric curves further reflect the areas of weight loss. The thermal degradation behavior also explains the fibrillation characteristics of BC and its purity, as indicated by the absence of any further degradation areas [73,74].

### 3.6. Antibacterial Activity of BC/CMC/Gly/Yeast Composite Films

**Figure 5:**
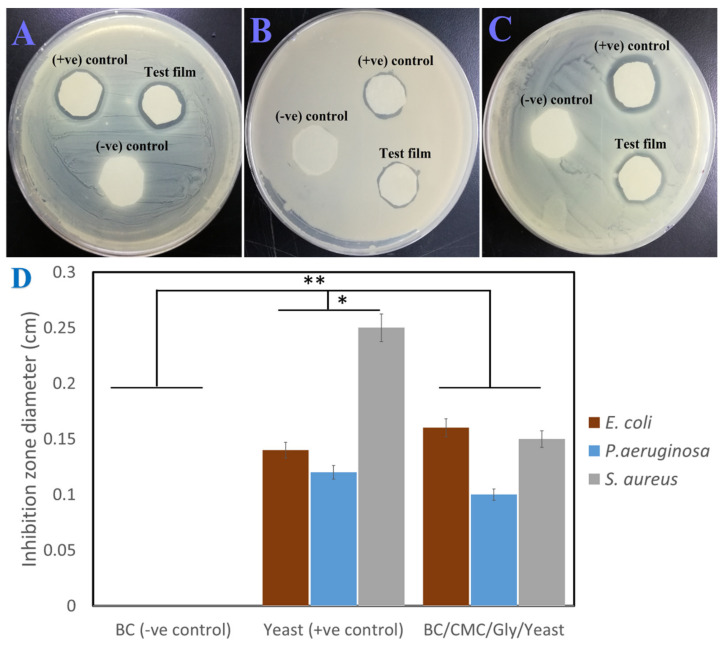
(**A**–**C**) Antimicrobial activity of BC/CMC/Gly/yeast composite films against Gram-positive (*S. aureus*) and Gram-negative bacteria (*P. aeruginosa* and *E. coli*) determined via disc diffusion method. A pure yeast extract and BC/CMC/Gly film were used as the positive control and negative control, respectively. (**D**) A quantitative presentation of inhibition zones produced by the sample and control against the tested microorganisms, * p < 0.05 and at ** p < 0.01.

The antibacterial activity of the BC/CMC/Gly/yeast composite film along with the positive control (i.e., yeast extract loaded on filter paper) and negative control (pure BC film) was evaluated against three bacterial strains: *E.coli*, *P. aeruginosa*, and *S. aureus*, via the disc diffusion method. These selected bacterial strains mainly cause diseases in humans and are responsible for food contamination and spoilage. The results of antimicrobial activity are shown in Figure 5, which shows that the negative control did not produce any inhibition zone against the selected bacterial strains, while the positive control produced inhibition zones of 14 mm, 12 mm, and 25 mm against *E. coli*, *P. aeruginosa*, and *S. aureus*, respectively, which are significantly higher than the negative control (** p < 0.01). In contrast, the BC/CMC/Gly/yeast composite film demonstrated antibacterial activity by producing clear inhibition zones of 16 mm, 10 mm, and 15 mm against *E. coli*, *P. aeruginosa*, and *S. aureus*, respectively, which was significantly higher than the negative control (** p < 0.01) and lower than the positive control (* p < 0.05). These results further demonstrate that the BC/CMC/Gly/yeast composite film possesses antibacterial activity against both Gram-positive (*S. aureus*) and Gram-negative bacteria (*P. aeruginosa* and *E. coli*). Previous studies have reported that both pristine BC [26] and CMC [64] do not possess any antibacterial activities, and these only show antibacterial activity when composited with other bactericidal elements such as metal nanoparticles [75,76], antimicrobial peptides [77], and some polymers such as chitosan [78]. Similarly, Gly is only used as the carbon source by different microorganisms and does not demonstrate any antibacterial activity [79]. Thus, it gives a clue that the antibacterial activity of BC/CMC/Gly/yeast composite films is solely due to yeast, which is in accordance with a previous report showing the antibacterial activity of *M. guilliermondii* or its metabolites and other yeast strains [34,35,80]. Furthermore, the antibacterial activity of *M. guilliermondii* or its metabolites was not greatly affected upon mixing with other additives such as BC, CMC, and Gly. The relatively low antibacterial activity of the BC/CMC/Gly/yeast composite film compared to the positive control could be attributed to the impregnation of yeast into the spaces in the film matrix which make it less accessible to interact with the bacterial cells and show bactericidal activity. These results demonstrate that the yeast-incorporated BC/CMC/Gly composite films could be used as the packaging material for extending the shelf life of different foods. In a previous study, a bread containing *M. guilliermondii* demonstrated a more prolonged shelf life than bread without yeast [34].

### 3.7. Biocompatibility of BC/CMC/Gly/Yeast Composite Films

**Figure 6:**
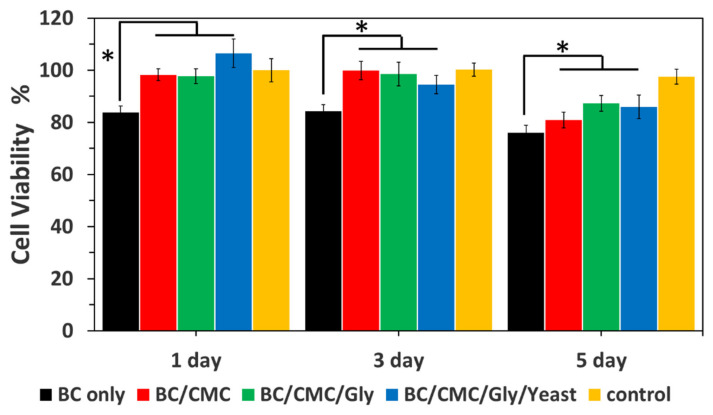
Viability of NIH-3T3 fibroblasts on pristine BC, BC/CMC, BC/CMC/Gly, and BC/CMC/Gly/yeast films after incubation for 1, 3, and 5 days, * *p* < 0.05. The absorption was recorded at 570 nm for all samples.

A biocompatible and non-toxic nature are the major requirements of any material to qualify its application in food packaging. These two factors have been widely studied in BC for its application in food, tissue engineering, drug delivery, and other biomedical applications [71]. The results of cell viability show that the cell growth on the composite films, both in the absence (BC/CMC and BC/CMC/Gly) and presence (BC/CMC/Gly/yeast) of yeast was significantly higher (* p < 0.05) than the pristine BC (Figure 6), indicating the non-toxic nature of yeast and other components of the composites on the viability of NIH-3T3 fibroblasts. After 24 h, the vitality of NIH-3T3 fibroblasts on pristine BC, BC/CMC, BC/CMC/Gly, and BC/CMC/Gly/yeast films was 82%, 96%, 95%, and 106%, respectively, which further increased to 84%, 100%, 97%, and 93% after 72 h. However, with further incubation up to 120 h, the viability of NIH-3T3 cells decreased to 73%, 80%, 86%, and 85% on pristine BC, BC/CMC, BC/CMC/Gly, and BC/CMC/Gly/yeast films, respectively. At all predefined points (i.e., 1, 2, and 3 days), the cell growth on the composite films was significantly higher than the pristine BC (* *p* < 0.05). The cell viability results demonstrate that the addition of yeast to the BC/CMC/Gly composite film did not cause any toxicity but rather enhanced the cell growth, thus indicating its non-cytotoxic and biocompatible nature. The results of cell viability at each time point indicate that the BC/CMC/Gly/yeast composite films have a higher OD value than the control and pristine BC, indicating their enhanced biocompatibility. Although the cell proliferation rate was similar in all groups after 3 days, the cells continued to grow on the composite films and showed a greater proliferation rate than the pristine BC after 5 days, indicating the enhanced biocompatibility of BC upon the addition of CMC, Gly, and yeast, which could be attributed to release of vitamins and minerals from yeast which could support the growth and proliferation of NIH-3T3 cells. These results are in agreement with previous reports [47,81]. The cell viability results further indicate that the selected 2 wt.% yeast for the preparation of the composite film is optimal and non-cytotoxic towards NIH-3T3 cells.

### 3.8. Real Packaging of Orange and Tomato with BC/CMC/Gly/Yeast Composite Films

**Figure 7:**
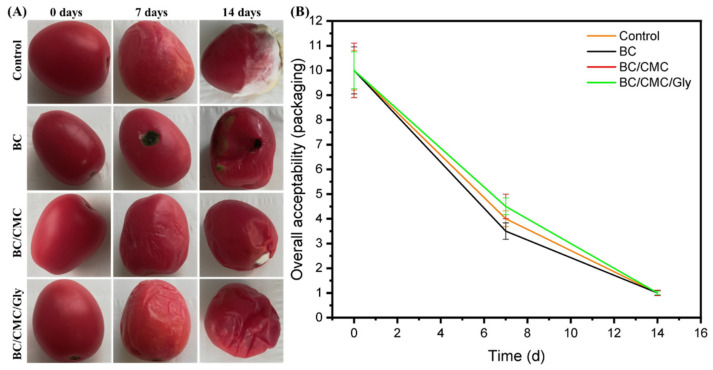
The results of preliminary analysis of (**A**) fruit coating and (**B**) evaluation of acceptance for the control (uncoated tomatoes) and pristine BC, BC/CMC, and BC/CMC/Gly films evaluated at 30 °C for two weeks.

Food packaging with antimicrobial materials has received immense consideration owing to its potential to prevent spoilage and enhance the shelf life of food [38]. The antimicrobial compounds or extracts with specific bioactivity are used to enhance the features of food packaging materials. In the preliminary studies, we evaluated the fruit packaging performance of pure BC, BC/CMC, and BC/CMC/Gly for two weeks at 30 °C; however, all such coatings showed similar results and did not provide any real protection to the fruits (Figure 7). Therefore, we only evaluated the fruit packaging performance of uncoated (control), BC-coated (Film-0), and BC/CMC/Gly/yeast-coated (Film-1) samples against tomatoes and oranges. After applying the BC/CMC/Gly/yeast packaging via the dipping method, the packaged food was preserved at four different temperatures: refrigerator (6 °C), room temperature (20 °C to 25 °C), and in the incubator (30 °C and 40 °C), for different time intervals. The food quality was observed visually by a panel of 10 judges and scored according to a predefined scale and recorded by taking photographs after different storage periods at respective storage temperatures.

**Figure 8:**
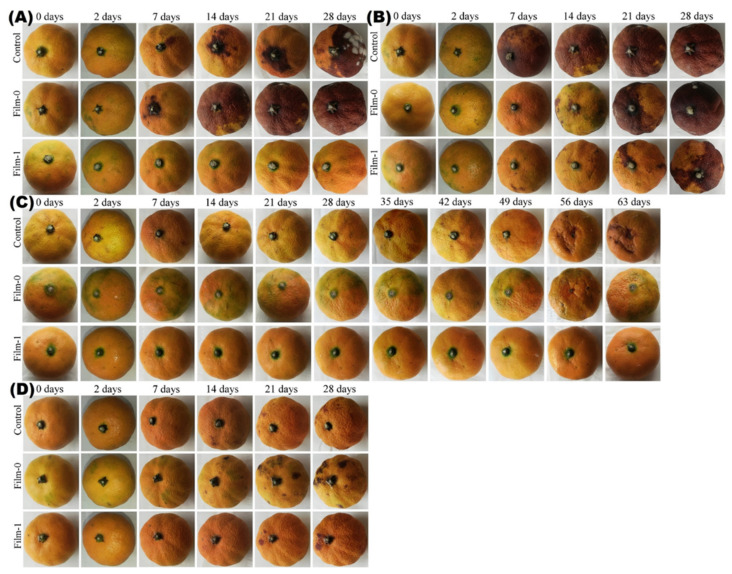
The photographs of uncoated oranges (control), BC-coated oranges (Film-0), and BC/CMC/Gly/yeast-coated oranges (Film-1) stored at (**A**) 30 °C, (**B**) 40 °C, (**C**) 6 °C, and (**D**) room temperature (20 to 25 °C) for different time intervals.

**Figure 9:**
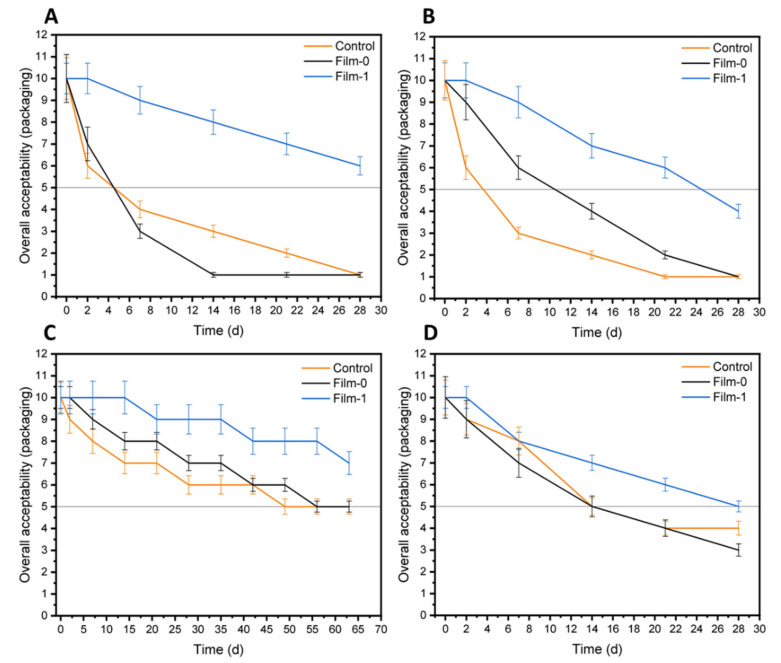
Evaluation of acceptance of uncoated oranges (control), BC-coated oranges (Film-0), and BC/CMC/Gly/yeast-coated oranges (Film-1) stored at (**A**) 30 °C, (**B**) 40 °C, (**C**) 6 °C, and (**D**) room temperature (20 to 25 °C for different time intervals.

The qualitative photographs of differently coated orange samples, including the control (uncoated), BC-coated (designated as Film-0), and BC/CMC/Gly/yeast-coated (designated as Film-1) preserved at different temperatures, are shown in Figure 8. The photographs show that at 30 °C (Figure 8A) and 40 °C (Figure 8B), both the control and Film-0 showed spots with brown color after 7 days and a drought-like appearance compared to the Film-1 after 28 days. Moreover, some sensory features such as odor and color were comparable among the control and test samples, although their quality changed. In contrast, no color or odor change was observed in Film-1-coated oranges stored at 6 °C for up to 63 days, while Film-0 showed a light drought after 14 days and continued to increase until 63 days (Figure 8C). In contrast, the control showed a little high drought under the same storage conditions. At room temperature (Figure 8D), both the control and Film-0 showed comparatively high drought after 28 days, while the film-1-coated oranges remained relatively fresh and preserved their quality after 28 days. Furthermore, the quantitative analysis of the results for sensory features such as the odor and color of oranges according to a predefined scale (1–2 = very poor, 3–4 = poor, 5–6 = fair, 7–8 = good, and 9–10 = excellent) and cutoff value (i.e., 5) for overall acceptability are shown in Figure 9 and Table 3. The results show that Film-1 demonstrated greater value than the cutoff, while the control and Film-0 demonstrated lower values than the cutoff at different treatment temperatures, except at 6 °C. Furthermore, the analysis of overall acceptability versus time analysis indicated that the value of Film-1-coated oranges remained above the cutoff value at all treatment temperatures, except 40 °C, after 28 days. In contrast, the uncoated and Film-0-coated samples remained fresh only for up to 14 days at room temperature. These quantitative results indicate that coating of oranges with BC/CMC/Gly/yeast greatly contributed to enhancing the shelf life for up to 14 additional days. Taken together, the findings of orange coating with BC/CMC/Gly/yeast as the packaging materials at different temperatures indicate an improved shelf life in the refrigerator for up to 63 days and acceptable storage for 28 days at room temperature and 30 °C. Further increasing temperature and extended incubation time damaged the food quality and odor. These results show that the BC/CMC/Gly/yeast composite could be a stable food packaging material for enhancing the shelf life of oranges at small and large scales. A previous study reported the development of coating materials comprising quaternized chitosan as an antibacterial agent and CMC for enhancing the morphological and physiological properties. The developed composite showed enhanced tensile strength, thermal stability, and water resistance and enhanced the shelf life of banana at 30 °C by showing antibacterial activity against *S. aureus* and *E. coli* [64]. In the present study, the demonstration of the antibacterial activity of the BC/CMC/Gly/yeast composite at various temperatures (6 °C, 20 to 25 °C, 30 °C, and 40 °C) provides a wider scope of storage conditions of the developed food packaging materials.

**Figure 10:**
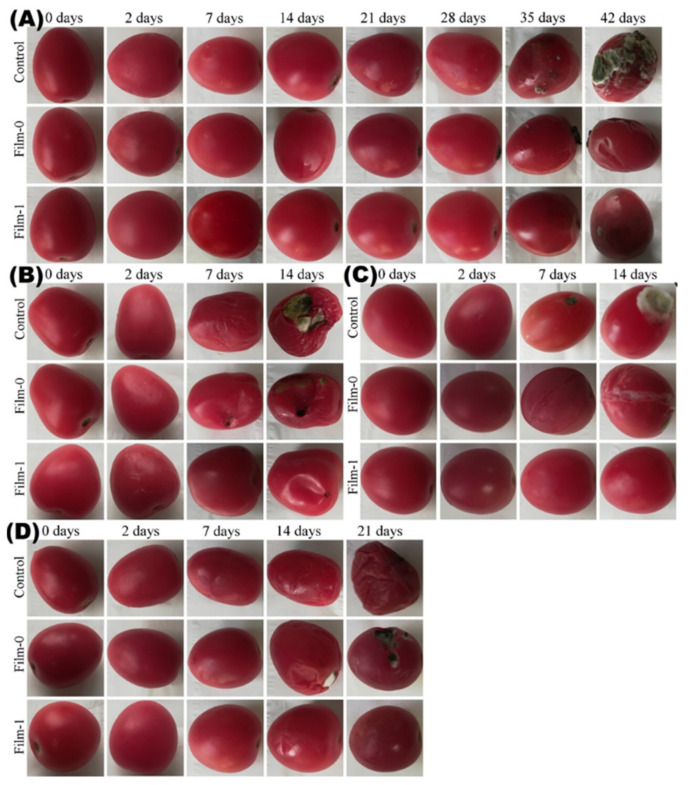
The photographs of uncoated tomatoes (control), BC-coated tomatoes (Film-0), and BC/CMC/Gly/yeast-coated tomatoes (Film-1) stored at (**A**) 6 °C, (**B**) 40 °C, (**C**) 30 °C, and (**D**) room temperature (20 to 25 °C) for different time intervals.

**Figure 11:**
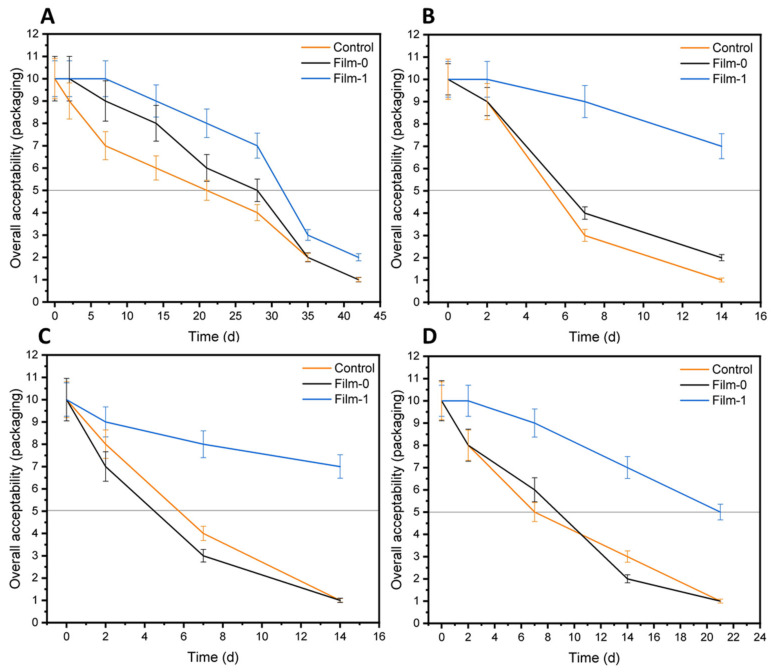
Evaluation of overall acceptability of uncoated tomatoes (control), BC-coated tomatoes (Film-0), and BC/CMC/Gly/yeast-coated tomatoes (Film-1) stored at (**A**) 6 °C, (**B**) 40 °C, (**C**) 30 °C, and (**D**) room temperature (20 to 25 °C) for different time intervals.

The qualitative results of tomato packaging with different samples and incubated for different time intervals at different temperatures are shown in Figure 10. The photographs show that Film-1 imparted high storage stability to tomatoes during the extended incubation at 6 °C (Figure 10A), 40 °C (Figure 10B), 30 °C (Figure 10C), and room temperature 20 to 25 °C (Figure 10D). In contrast, the control and Film-0 showed medium rot after 3 weeks at 6 °C. The tomatoes started to become spoiled after 6 days when stored at 30 °C, 40 °C, and room temperature. The quantitative analysis of the results for sensory features such as odor and color of tomatoes according to a predefined scale (1–2 = very poor, 3–4 = poor, 5–6 = fair, 7–8 = good, and 9–10 = excellent) and cutoff value (i.e., 5) for overall acceptability are shown in Figure 11. At room temperature, the uncoated and Film-0-coated samples only remained fresh for 7 days, while the Film-1-coated samples remained fresh for 21 days, indicating an increased shelf life of 14 days upon packaging with BC/CMC/Gly/yeast. Furthermore, the Film-1-coated tomatoes also demonstrated high overall acceptability at other storage temperatures (i.e., 6 °C, 30 °C, and 40 °C). These results indicate that similar to orange packaging, the packaging of tomatoes with BC/CMC/Gly/yeast resulted in acceptable packaging performance for extended storage, not only at 6 °C but even at room temperature and 30 to 40 °C for up to two weeks [64].

Overall, the results of fruit coating experiments demonstrate that the packaging with BC/CMC/Gly/yeast greatly contributed to protecting the texture of oranges and tomatoes against environmental factors and preserved their odor and color for an extended period of time.

## 4. Conclusions

The developed BC/CMC/Gly/yeast composite films demonstrated good water solubility and flexibility and showed good antibacterial activity against Gram-positive and Gram-negative bacteria, indicating their suitability for packaging applications. The developed composite films were non-cytotoxic towards NIH-3T3 fibroblasts, indicating their usefulness and safe nature for packaging application. The coating of oranges and tomatoes, as real food samples, with the BC/CMC/Gly/yeast composite film enhanced their shelf life by protecting their texture against environmental factors and preserving their odor and color. All these findings provide a base for the development of edible food packaging materials for the storage of sensitive food materials.

## Figures and Tables

**Table 1:** Water solubility and moisture content of different composite films.

Films	Moisture Content—WC (%)	Water Solubility—WS (%)
BC/CMC	9.72 ± 0.32	22.28 ± 1.44
BC/CMC/Gly	30.11 ± 1.90	39.54 ± 2.30
BC/CMC/Gly/yeast	23.66 ± 1.59	42.86 ± 2.78

Values are given as mean ± standard deviation.

**Table 2:** Mechanical properties of pristine BC and BC-based different composite films.

Films	Tensile Strength (MPa)	Elongation at Break (%) ^a^
BC	17.02 ± 1.19	4.77 ± 0.56
BC/CMC	19.64 ± 1.43	4.61 ± 0.61
BC/CMC/Gly	5.01 ± 0.32	22.96 ± 1.24
BC/CMC/Gly/yeast	2.23 ± 0.33	15.53 ± 0.84

**^a^** Elongation at break (%) calculated using Equation (3).

**Table 3:** Values of the acceptability degree of oranges and tomatoes for uncoated (control), BC-coated (Film-0), and BC/CMC/Gly/yeast-coated (Film-1) under different storage conditions, including at 6 °C, 30 °C, 40 °C, and room temperature (20 to 25 °C), for a different time interval.

Sample	Temperature (°C)	Oranges		Tomatoes	
		Minimum Accepted Value w.r.t Sensory Features	Minimum Accepted Value w.r.t Time (Days)	Minimum Accepted Value w.r.t Sensory Features	Minimum Accepted Value w.r.t Time (Days)
**Control**	6	5 ± 0.35	49	5 ± 0.45	21
	20–25	5 ± 0.4	14	5 ± 0.42	7
	30	6 ± 0.57	2	9 ± 0.81	2
	40	6 ± 0.54	2	8 ± 0.64	2
**Film-0**	6	5 ± 0.25	56	5 ± 0.5	28
	20–25	5 ± 0.47	14	6 ± 0.54	7
	30	7 ± 0.77	2	9 ± 0.63	2
	40	6 ± 0.54	7	7 ± 0.66	2
**Film-1**	6	7 ± 0.52	63	7 ± 0.56	28
	20–25	5 ± 0.25	28	5 ± 0.35	21
	30	6 ± 0.42	28	7 ± 0.56	14
	40	6 ± 0.48	21	7 ± 0.52	14

Control: uncoated fruit samples; w.r.t: with respect to; sensory features: odor, color, dryness, and contamination.

## Data Availability

No new data were created or analyzed in this study. Data sharing is not applicable to this article.
